# Successful Retrieval of a Torn KUSABI Trapping Balloon Catheter From the Coronary Artery and Verification of the Reproducibility of Trapping Balloon Catheter Shaft Tears

**DOI:** 10.7759/cureus.58508

**Published:** 2024-04-18

**Authors:** Morio Ono, Toshitaka Okabe, Naoei Isomura, Masahiko Ochiai

**Affiliations:** 1 Division of Cardiology, Showa University Northern Yokohama Hospital, Yokohama, JPN

**Keywords:** balloon deflation, rupture, complication, kusabi catheter, balloon rupture

## Abstract

The balloon trapping technique is frequently used during percutaneous coronary intervention, which is a common treatment for ischemic heart disease. A 68-year-old man with induced ischemia, stenotic lesions, and arterial calcifications underwent catheterization of the circumflex artery and debulking of lesions. During the removal of the catheter, the tip of the balloon catheter used in the procedure dislodged and entered the circumflex artery. After successfully retrieving the catheter, we conducted a bench test of the balloon catheter to determine the cause of the tear. The results suggested that the tearing of the KUSABI balloon might have been caused by manual pulling of the shaft quickly at an inflation pressure of 14 atm and that twisted wires were not involved in balloon tearing. The tensile strength of the balloon catheter was 5N. We believe that the balloon tore owing to excessive force applied to dislodge the tip and because the trapping balloons were not properly deflated. As KUSABI trapping balloons have had a rupture rate of just 0.003% since their launch in 2013, we recommend paying attention to KUSABI balloon deflation within the guiding catheter before its retrieval in order to ensure that only a gentle pull is needed. If resistance is felt during the removal of the KUSABI balloon, it should be confirmed that the tip is in place after removing it.

## Introduction

Percutaneous coronary intervention (PCI) is a common treatment for ischemic heart disease. In complex PCI procedures, micro-catheter exchanges using the balloon trapping technique with trapping balloon catheters, such as the KUSABI catheter (Kaneka Medical Products, Osaka, Japan), are frequently used. The KUSABI catheters are simple and user-friendly devices for micro-catheter exchange [1]. Although tearing of conventional balloon catheters has been reported, to the best of our knowledge, tearing of the KUSABI trapping balloon has not been reported [2,3].

Herein, we report our experience with a KUSABI catheter that tore between the shaft and body of the balloon and entered the coronary artery. We successfully removed the intravascular foreign body (the torn balloon) from the coronary artery with a snare. Additionally, we report the results of bench-testing the tear resistance of the KUSABI catheter.

## Case presentation

A 68-year-old man presented to our hospital with shortness of breath upon exertion. Myocardial perfusion imaging using thallium-201 revealed ischemia in the anterior and posterior walls. Coronary angiography revealed severe stenotic lesions and calcifications in the proximal left anterior descending (LAD) and middle circumflex (CX) arteries. As the Syntax score was low at zero points and because of the patient’s preference, we planned to perform PCI of LAD and CX.

April 21, 2015. A 7-Fr Hyperion SPB 4.0 (Asahi Intecc Co., Seto, Japan) was inserted into the left coronary artery (LCA) via the right radial artery. First, the lesions in the CX were treated. A Promus Premier stent (diameter, 2.5 mm; length, 38 mm; Boston Scientific, Marlborough, MA, USA) was implanted successfully after an intravascular ultrasound (IVUS) investigation. Subsequently, a Sion Blue guidewire (Asahi Intecc) was crossed with the LAD using a Caravel micro-catheter (Asahi Intecc). The caravel was removed using the trapping method with a KUSABI catheter (Kaneka Medical Products).

**Figure 1:**
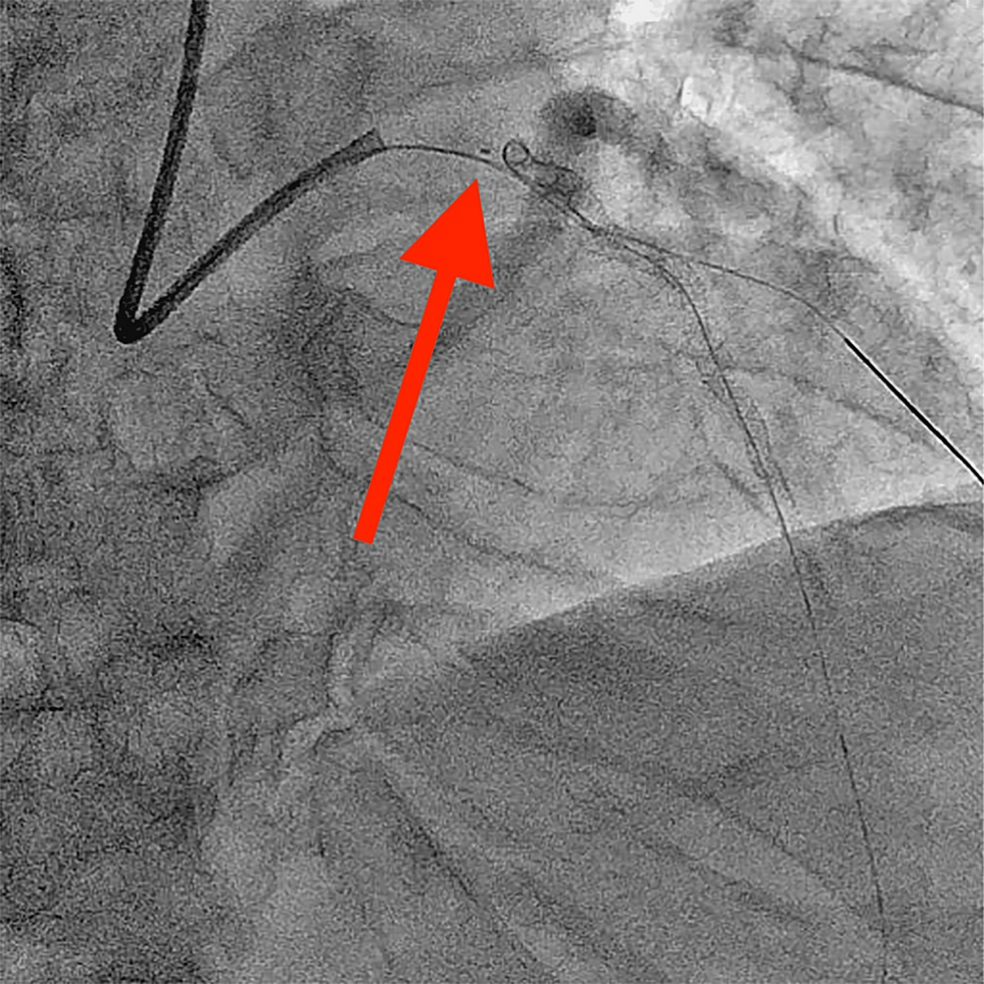
The tip of the torn KUSABI advanced into the left main coronary trunk via coronary angiography.

**Figure 2:**
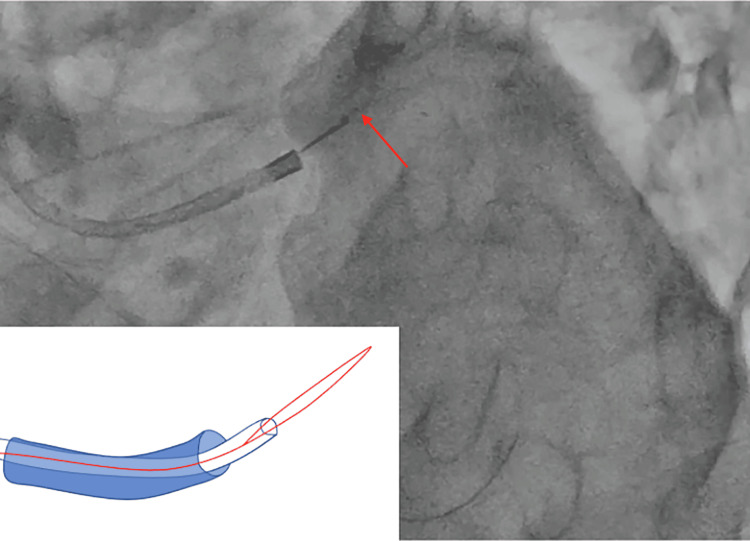
Successful retrieval of the tip of the KUSABI catheter using a gooseneck snare. Image at the time of retrieval. Image credit: Morio Ono

As the IVUS catheter could not cross the lesion, we decided to debulk the calcified lesion. The Sion Blue guidewire was replaced with a ROTAWIRE Floppy guidewire (Boston Scientific) using Caravel and KUSABI catheters. A rotational atherectomy was performed. After debulking the lesion, the patient complained of chest pain, and electrocardiography revealed ST elevation in the precordial leads. Coronary angiography revealed a slow-flow phenomenon in the LAD. After dilation of the lesion with a non-compliant balloon, the forward flow was restored, and the ST elevation improved. A Promus Premier stent (diameter, 2.5 mm; length, 32 mm) was delivered using a guide-extension catheter and was placed using 18 atm pressure in the middle LAD. A Sion guidewire (Asahi Intecc) was advanced through the first diagonal branch using a Crusade dual-lumen catheter (Kaneka Medical Products). The KUSABI balloon catheter was inflated to 8 atm for trapping and removing the crusade catheter. The assistant felt resistance when pulling the KUSABI catheter out of the guiding catheter (GC), but we did not check the catheter tip. After removing the crusade catheter, coronary angiography revealed that the tip of the KUSABI catheter with a marker got dislodged in the left main trunk (Figure 1). We unsuccessfully attempted to retrieve this foreign body into the GC with a dilated balloon (diameter, 2.5 mm; length, 15 mm). Moreover, the tip of the KUSABI catheter moved into the CX. The patient experienced chest pain and ST elevation in the precordial leads. As angiography revealed a recoiled lesion of the LAD, a Promus Premia stent (diameter, 3.0; length, 20 mm) was implanted into the LAD. The tip of the KUSABI catheter was successfully captured and removed using a 4-mm Amplatz gooseneck snare (ev3 Endovascular, Plymouth, MN, USA) (Figure 2, Video 1). Post-dilatation and kissing balloon inflation of the LAD and diagonal branches were performed. The procedure was completed without any complications. 

**Video 1:** Snare catch Torn KUSABI was caught with a snare and extracted.

**Figure 3:**
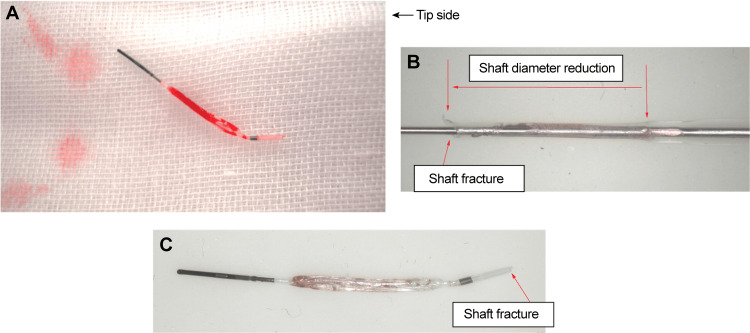
A: Tip of the retrieved KUSABI balloon catheter. B: Shaft diameter reduction and deformation of the retrieved ruptured KUSABI balloon catheter. C: Tip-side shaft fracture of the retrieved KUSABI balloon catheter.

We encountered the case of a trapping balloon catheter tear, which is a rare complication. The KUSABI catheter was inspected after its removal (Figure 3). Additionally, we performed a bench test on the tear resistance of the KUSABI catheter to confirm that this phenomenon could be reproduced.

Confirmation of the manufacturing records

Kaneka Medical Products measured the tensile strength of each production lot, and we confirmed that the tensile strength of the lot was 5 N (standard: ≥3 N) and that the balloon passed the test.

Reproduction experiment

Tensile Strength Test While Inflated in 6-8 French GCs

**Figure 4:**
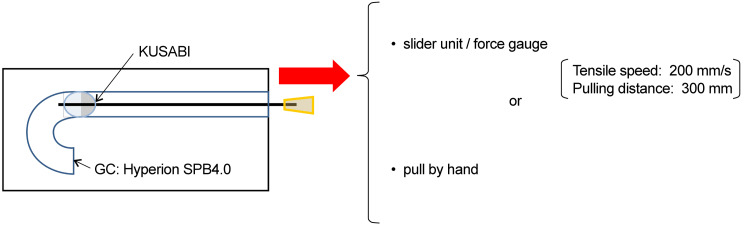
Schematic diagram of the reproduction experiment Image credit: Morio Ono

Methods: Tensile strength tests were conducted with a slider or manually using samples inflated with a rated burst pressure of 14 atm in 6-, 7-, and 8-Fr GCs. The KUSABI balloon catheter did not burst when pulled by hand after applying 8 atm pressure. The samples consisted of KUSABI, KUSABI Long, and IKAZUCHI Zero (all from Kaneka Medical Products) balloon catheters (Figure 4).

Results: When pulling with the slider unit, the shaft either emerged from the GC as it was broken or broke after the inflation of the balloon. The mode of breakage was different from that of the returned product. The rupture mode, which was the most similar to that in the present case, was observed when the shaft was manually pulled quickly at an inflation pressure of 14 atm. The IKAZUCHI Zero balloon ruptured after inflation, and its maximum tensile load at the time of rupture was lower than that of the KUSABI balloon alone. No difference was observed in the size of the GC or the number of inflations (Table 1).

**Table 1:** Results of reproduction experiments GC: Guiding catheter

GC	Sample	Rupture	Maximum tensile load (N)	Tensile condition	expansion pressure (atm)	Expansion times	Condition of shaft after tension
6Fr	KUSABI	(−)	14.4	Slider unit/force gauge	14	1	Torsion of the shaft
6Fr	KUSABI	(−)	-	Hand	14	1	Expansion of the shaft
6Fr	KUSABI	(−)	-	Hand	14	1	Expansion of the shaft
6Fr	KUSABI	(−)	10.6	Slider unit/force gauge	14	1	Torsion of the shaft
7Fr	KUSABI	(−)	13.7	Slider unit/force gauge	14	1	Torsion of the shaft
7Fr	KUSABI	(−)	15.1	Slider unit/force gauge	14	10	Torsion of the shaft
7Fr	KUSABI	(−)	-	Hand	8	1	No change
7Fr	KUSABI	(−)	-	Hand	8	1	No change
7Fr	KUSABI	(+)	-	Hand	14	1	Reproducibility similar to our case
7Fr	KUSABI	(+)	-	Hand	14	1	Reproducibility similar to our case
7Fr	KUSABI	(+)	-	Hand	14	1	Reproducibility similar to our case
7Fr	KUSABI	(+)	-	Hand	14	1	Intermediate stretch
7Fr	KUSABI	(+)	-	Hand	14	1	Intermediate stretch
7Fr	KUSABI	(+)	-	Hand	14	10	Reproducibility similar to our case
7Fr	KUSABI	(+)	-	Hand	14	1	Fracture near hypo connection
7Fr	KUSABI long	(+)	14.7	Slider unit/force gauge	14	1	Balloon weld fracture
7Fr	KUSABI long	(+)	14.6	Slider unit/force gauge	14	1	Fracture near hypo connection
7Fr	IKAZUCHI Zero	(+)	7.6	Slider unit/force gauge	14	1	Intermediate stretch and fracture near hypo connection
7Fr	IKAZUCHI Zero	(−)	7.5	Slider unit/force gauge	14	1	Intermediate stretch
7Fr	IKAZUCHI Zero	(+)	-	Hand	14	1	Intermediate stretch and fracture near hypo connection
8Fr	KUSABI	(−)	16.7	Slider unit/force gauge	14	1	Shaft extension
8Fr	KUSABI	(−)	16.7	Slider unit/force gauge	14	10	Shaft extension

## Discussion

The frequency of rupture of the KUSABI trapping balloon has been 0.003% since its launch in 2013. In the present case, based on the bench test results, the tearing of the KUSABI balloon might have been caused by an excessive pulling force, probably because the trapping balloons were not properly deflated. The small-balloon technique, the two-wire technique, and the use of snares and forceps have been reported as methods for retrieving intra-vascular foreign bodies [4]. The small balloon technique is simple and easy to perform. A coronary wire is advanced distally into the foreign body. A small compliant coronary balloon is mounted on the coronary wire, inflated to low pressure, and then pulled back over the coronary guidewire. If the procedure is successful, the foreign body can be pulled back into the GC. Generally, this approach is attempted first because there are no additional tools to be added [4]. In two small case series, the success rate of the small balloon technique for retrieving lost stents ranged from 50% to 80% [5,6]. In the present case, the small balloon technique was initially attempted but was unsuccessful. We used a snare to successfully retrieve a foreign body from the coronary artery. A snare is alternatively used for retrieving a foreign body from a coronary or peripheral artery. Snares of various sizes and shapes suitable for different vessels and types of foreign bodies are available. Various snare techniques for retrieving intra-vascular foreign bodies have been devised, such as the loop snare proximal grab technique and loop snare lateral grasp technique. In the present case, as the severed end of the torn shaft was sharp and hard, there was a high risk of vascular injury. In the case of a torn shaft, it is best to grasp the severed end of the shaft straight against the GC and retrieve it directly from the GC [7]. Goksin et al. reported that approximately half of the patients in whom the guidewire or GC (contrast, balloon, or IVUS) was difficult to remove during PCI underwent surgery, and there are scattered reports of surgical procedures both in Japan and overseas [8]. To the best of our knowledge, there have been no case reports of shaft rupture of a trapping balloon or similar case reports of shaft ruptures of ultrahigh-pressure balloons [9] or IVUS ruptures [10]. Since November 2016, the balloon length has varied from 10 to 15 mm. However, the shaft design has not been changed, and attention should be paid to KUSABI balloon deflation within the GC before its retrieval. From the results of our bench test and the situation, we considered that the KUSABI balloon had ruptured due to the pulling of the catheter by the assistant, even though it had not been deflated. The balloon of the KUSABI catheter can rupture after 8 atm dilatation with 7Fr GC. On the contrary, in in-vitro experiments, the balloon of the KUSABI catheter did not burst when pulled by hand after 8 atm pressure with a 7Fr GC but did reproducibly burst at 14 atm. We attributed this to the fact that our bench test was performed in vitro, and the backup force of the GC was also relevant in the actual case. A gentle pull is a feasible approach to avoid tearing of the KUSABI balloon.

## Conclusions

We experienced a rare case of trapping balloon rupture and determined the cause of the rupture in a reproduction experiment. The failure in this case comprised not only the rupture of the KUSABI balloon but also the late detection of this rupture. Following coronary angiography, the KUSABI balloon had strayed into the coronary artery. If we had detected the rupture of the KUSABI balloon before coronary angiography, we could have used the small balloon technique to remove together with the GC the KUSABI balloon that remained in the GC. Based on this experience, we recommend paying attention to the deflation of the KUSABI balloon within the GC before its retrieval to avoid excessive force on the trapping balloon. Operators should check Kusabi after pulling out from the guide catheter.
